# Glutamic Acid Decarboxylase 1 Gene Methylation and Panic Disorder Severity: Making the Connection by Brain Gray Matter Volume

**DOI:** 10.3389/fpsyt.2022.853613

**Published:** 2022-05-24

**Authors:** Huiqin Wu, Yuan Zhong, Huazhen Xu, Huachen Ding, Shiting Yuan, Yun Wu, Gang Liu, Na Liu, Chun Wang

**Affiliations:** ^1^Nanjing Brain Hospital Affiliated to Nanjing Medical University, Nanjing, China; ^2^School of Psychology, Nanjing Normal University, Nanjing, China; ^3^Cognitive Behavioral Therapy Institute of Nanjing Medical University, Nanjing, China

**Keywords:** panic disorder, glutamic acid decarboxylase 1, gray matter volumes, DNA methylation, mediation effect

## Abstract

**Objective:**

This study aimed to test the hypothesis that the relationship between glutamic acid decarboxylase (GAD) 1 gene methylation and severity of clinical symptoms of panic disorder (PD) is mediated by the effect of GAD1 gene methylation on gray matter volume (GMV) and the effect of GMV on PD.

**Methods:**

Panic disorder (*n* = 24) patients were recruited consecutively from the Affiliated Brain Hospital of Nanjing Medical University through outpatient and public advertising, eligible healthy controls (HCs) (*n* = 22) were recruited from public advertising. We compared GMV and GAD1 gene methylation in PD and HCs to estimate the differences, and on the basis of the relationship between gray matter volumes and GAD1 gene methylation in PD patients was evaluated, the role of GMV as a mediator of GAD1 gene methylation and PD clinical symptoms was analyzed.

**Results:**

Panic disorder patients had significantly lower methylation in the GAD1 promoter region on Cytosine-phosphate-guanine (CPG) 7 than HCs (*t* = 2.380, *p* = 0.021). Pearson correlation analysis found a significant negative association between cg171674146 (cg12) site and clinical severity (*n* = 24, *r* = −0.456, *p* = 0.025). Compared to HCs, patients with PD had decreased gray matter volumes in several brain regions, which were also associated with PD severity. Left postcentral gyrus (PoCG) GMV mediated the association between cg12 methylation and PD severity, and there was a significant mediation effect of right angular gyrus (ANG) gray matter volumes on the relationship between cg12 methylation and PD severity.

**Limitation:**

No direct results can be derived for methylation patterns in different brain regions; the study is cross-sectional; relatively small size.

## Introduction

Panic disorder (PD) is defined as episodic, unexpected panic attacks with no clear trigger that includes worry about further attacks and modifications to behavior in maladaptive ways to avoid them (1). Panic disorder is a common mental illness with a lifetime prevalence rate of 0.5%; the China Mental Health Survey reports estimates a 12-month prevalence of 0.3% (2).

The pathogenesis of PD is complex, comprised of an interaction of biological factors, particularly genetic factors (heritability estimates: 48%) (3) and brain structure (4). Gamma-aminobutyric acid (GABA) is the main inhibitory neurotransmitter of the central nervous system, and its dysfunction is considered to be one of the main neurobiological pathomechanisms of anxiety (5), especially patients with anxiety and panic disorder (6). GAD is the key rate-limiting enzyme that catalyzes the decarboxylation of glutamate to synthesize Gamma -aminobutyric acid, it has two isozyme forms–GAD67 (GAD1) and GAD65 (GAD2). The GAD1 gene is the rate-limiting enzyme for glutamate synthesis of GABA in the brain, GAD2 gene is present in membranes and nerve endings, so it is more sensitive to the effects of GABA levels (7). GAD’s role in the pathogenesis of PD has been well-established. One study analyzed the association of the GAD1 and GAD2 genes with genetic risk for a range of diseases. They found that variations in the GAD1 gene may affect susceptibility across a range of anxiety disorders (8). Another study suggested that patients with panic disorder exhibited significantly lower average GAD1 methylation than HCs, though no methylation alterations were observed for the GAD2 gene (9). A third study tested the genetic association of 93 single nucleotide polymorphisms (SNPs) with anxiety disorders in the Finnish population-based Health 2000 sample (282 cases and 575 matched controls), they showed that several SNPs in the GAD1 gene (rs769401, rs3791851, and rs769395) were associated with PD, with GAD1 having the most obvious association (10). Gorman (11) proposed a fear network model (FNM) of panic disorder based on an animal model of fear. FNM is centered in the amygdala and includes the thalamus (THA), hypothalamus, and hippocampus, as well as the locus ceruleus, periaqueductal gray area, and other brainstem sites. However, more extended regions of FNM have been identified in recent imaging studies. For example, Lai (12) suggests FNM also includes sensory regions of occipital lobe, temporal cortex, insula, and parietal cortex.

A previous meta-analysis of PD conducted by our research group revealed significant volume reductions in the right insula [extending to the PoCG, right inferior frontal gyrus, rolandic operculum, superior temporal gyrus (STG) and putamen], median cingulate/paracingulate gyrus, and SFG (13). In addition, some researchers have reported significant volume reductions in parietal regions (14) and the THA (15) in PD patients, whereas significant increases in volume have been found in the left inferior frontal gyrus (IFG), midbrain (16) and cuneus (17). The PoCG has been associated with functions of receiving, integrating, and interpreting most of the sensory information in the human body (18). Gorman et al. (11) showed that abnormalities in PoCG could potentially lead to misinterpretation of somatosensory information, thus allowing inappropriate activation of the FNM through misleading excitatory inputs to the amygdala (11). Another region that has gained attention is the ANG. ANG is important in empathic response, emotional regulation, mood, anxiety, and is associated with meditation and calmness (19). Previous studies reported that not only do PoCG and ANG play an important role in panic disorder, a correlation between GMV of those regions and clinical symptoms in PD exists as well (20).

Although evidence suggests that epigenetic alterations of GAD1 gene and gray matter volumes alterations of PoCG and ANG play an important role in the onset and maintenance of panic disorder, whether GAD1 methylation influences GMV of PoCG and ANG in PD has not yet been confirmed. Thus, the current study sought to explore the differences in whole brain gray matter volume and GAD1 gene promoter methylation levels between patients with panic disorder and the normal population. And furthermore, to explore the correlation between GAD1 methylation, brain gray matter volume and Panic Disorder Severity Scale (PDSS) scores. We hypothesized that GMV of PoCG and ANG mediates the relationship between GAD1 methylation and panic disorder severity.

## Materials and Methods

### Participants

This study was approved by the Ethics Committee Institute of the Brain Hospital of Nanjing Medical University, and all participants will receive a detailed explanation of the process before signing the written informed consent.

Panic disorder patients were recruited consecutively from the Affiliated Brain Hospital of Nanjing Medical University through outpatient and public advertising. The inclusion criteria for patients in the PD group were as follows: (1) A diagnosis of PD by a psychiatrist according to the fourth edition of the DSM (Diagnostic and Statistical Manual of Mental Disorders); (2) Filter by Mini-international Neuropsychiatric Interview Chinese version; (3) Ability to read and write Chinese at least in 6th grade, right handedness; (4) 18–55 years old; (5) Cooperation with psychological tests and completion of questionnaires. Exclusion criteria were: (1) Any other psychiatric illnesses or disorders of the nervous system; (2) Any serious comorbidity; (3) Any psychotherapy or medical treatment in the past 6 months; (4) Pregnancy and/or breastfeeding; (5) Inability to complete MRI. In HC group. The inclusion criteria for patients were as follows: (1) 18–55 years old; (2) Hamilton Anxiety Rating Scale (HAMA) score ≤ 7; (3) Right handedness; (4) Ability to complete all examinations. Exclusion criteria were: (1) Any other psychiatric illnesses or disorders of the nervous system; (2) Any serious comorbidity; (3) Any psychotherapy or medical treatment in the past 6 months; (4) Pregnancy and/or breastfeeding; (5) Inability to complete MRI.

### Measure

A self-report questionnaire was used to collect demographic data on the subjects, including: gender, age, years of education, duration of illness, ethnicity, contact information, history of psychological counseling, history of physical illness, and so on. The PDSS (21) was used to assess panic symptom severity for patients with PD. The HAMA (22) is a well-established reliability and validity instrument used to assess the severity of anxiety for each subject.

### DNA Methylation Data

After obtaining informed consent, 5 ml of peripheral blood was retained from all subjects and stored in a refrigerator at −80°C. Blood samples that passed DNA quality testing were subjected to DNA sequencing. Based on the software primer3, amplification was performed using the technique of multiplex PCR, using the standard human genome as a template. The samples were processed using EZ DNA Methylation-Gold Kit reagent to convert genomic DNA unmodified by methylation from cytosine C to uracil U, and multiplexed PCR amplification was performed. High-throughput sequencing was performed in a 2 × 150 bp double-end sequencing mode using the Illumina Hiseq platform for detection of GAD1 methylation levels and analysis of the results.

### MRI Acquisition and Processing

While waiting for the scans, the researcher communicated with the subjects about precautions and reminded them to remove metal-type jewelry. During the scans, each subject was asked to remain still and close their eyes, use foam pads on both sides of their head to reduce head movement, and wear earplugs to protect their hearing. The magnetic resonance scanning involved in this study used the latest ultra-high field VerioMRI machine (magnetic field strength 3.0T, gradient field strength 45mT, and switching rate 150mT) from the Department of Radiology, Affiliated Brain Hospital of Nanjing Medical University. The sagittal images cover the entire brain with the following parameters: field of view (FOV) = 240 × 240 mm, repetition time (TR)/echo time (TE) = 1900/2.48 ms, matrix size = 128 × 128, slice thickness = 3 mm, interslice gap = 0.5 mm.

In order to minimize the errors caused by the data acquisition process, the CAT12 software package (C. Gaser, Structural Brain Mapping group, Jena University Hospital, Jena, Germany) based on the MATLAB platform was used to perform a series of original images of the 3D structure image. The preprocessing program was performed according to the following protocol: (1) Use the MATLAB platform-based SPM12 (Statistical Parametric Mapping) software package^1^ to view the 3D structure image of each subject, and manually rotate and re-create the image orientation to ensure that the structural images of each subject are aligned in space; (2) Remove the unevenness and noise of the MRI scanning process to normalize the image; (3) Based on the DARTEL algorithm, register all images to the default Template; (4) Divide the image after spatial normalization into three components of white matter, gray matter and cerebrospinal fluid (23), and calculate the total intracranial volume (TIV). This segmentation method used partial volume estimation (PVE) to deal with partial volume effects (24); (5) Use the CAT12 quality inspection process to evaluate the quality of the gray matter image after segmentation to ensure the subsequent data analysis process is based on good data quality; (6) Use a smoothing kernel (full width at half maximum, FWHM) of 8 mm to smooth all gray matter images.

### Statistical Analysis

This study used SPSS 22.0 software to analyze the general demographic data and questionnaire data, the difference in gender between PD patients and HCs was estimated with Chi square test, and an independent *t*-test was utilized to determine the potential differences in age, education duration, duration of illness, HAMA scores, and GAD1 methylation between PD patients and HCs. SPSS 22.0 was used to calculate the Pearson correlation between differentially methylated sites of GAD1 gene and PDSS scores. Differential analysis of whole brain gray matter volume between PD and HCs was performed using SPM 8, and no covariates were included because none of the demographic variables were significantly different. The minimum cluster size was 50 voxels, and differences were considered significant if *p* < 0.05. Multiple regression analysis using SPM8 was used to calculate the correlation between gray matter volumes and GAD1 methylation sites in PD patients, as well as the correlation between PDSS scores and GMV in panic disorder patients, with no covariate. Based on the above two correlations, the correlation loci were extracted using the image caculator in REST1.8 with the formula i1. *i2. Simple mediation analyses were performed using PROCESS, no covariates in the model. The Model4 intermediary model was constructed using the PROCESS plug-in in SPSS 22.0 software based on the Bootstrap method (25). The methylation level of GAD1 gene was used as the independent variable (X), the gray matter volume of each brain region was the mediating variable (M), and the total PDSS score was the dependent variable (Y). All analyses are based on a bootstrap sample of 5,000 Q20, and the mediation effect is considered significant when the confidence interval (CI) does not include zero (26).

## Results

### Demographic and Clinical Characteristics

We recruited 24 patients with PD (age ± SD: 31.6 ± 6.8, 12 males) and 22 HCs (age ± SD: 33.1 ± 7.1, 11 males) in this study, and all signed informed consents. The demographic and clinical characteristics of participants are presented in Table 1. Sex, age, and education did not differ among groups (all *p* > 0.05), whereas differences in HAMA total scores did (*p* < 0.001). The cumulative illness duration of the patients was 2.9 ± 3.6 years (Table 1). The average PDSS score in patients was 16.6 (*n* = 24, SD = 2.2), and ranged from 9 to 20. Eight patients did not complete the clinical measurement completely; 46 subjects had DNA methylation data, and 16 patients and 10 HCs had neuroimaging data.

**TABLE 1:** Demographic data of patients with panic and healthy controls.

	Patients	Controls	*p*-values
Gender(number)	M (12)/F (12)	M (11)/F (11)	0.087^a^
Age, mean (SD), years old	31.6 (6.8)	33.1 (7.1)	0.466^b^
Education duration, mean (SD), years	13.9 (3)	15.6 (3.8)	0.110^b^
Duration of illness, years	2.9 (3.6)	–	<0.001^b^
HAMA	20.6 (7.7)	2.2 (2.9)	
PDSS	16.6 (2.2)	–	

*a = χ^2^ b = independent sample t-test. SD, standard deviation; F, female; M, male; HAMA, Hamilton rating scale for anxiety; PDSS, panic disorder severity scale.*

### Lower Methylation in the Promoter of Glutamic Acid Decarboxylase 1 Gene in Panic Disorder Compared With Healthy Controls

**FIGURE 1:**
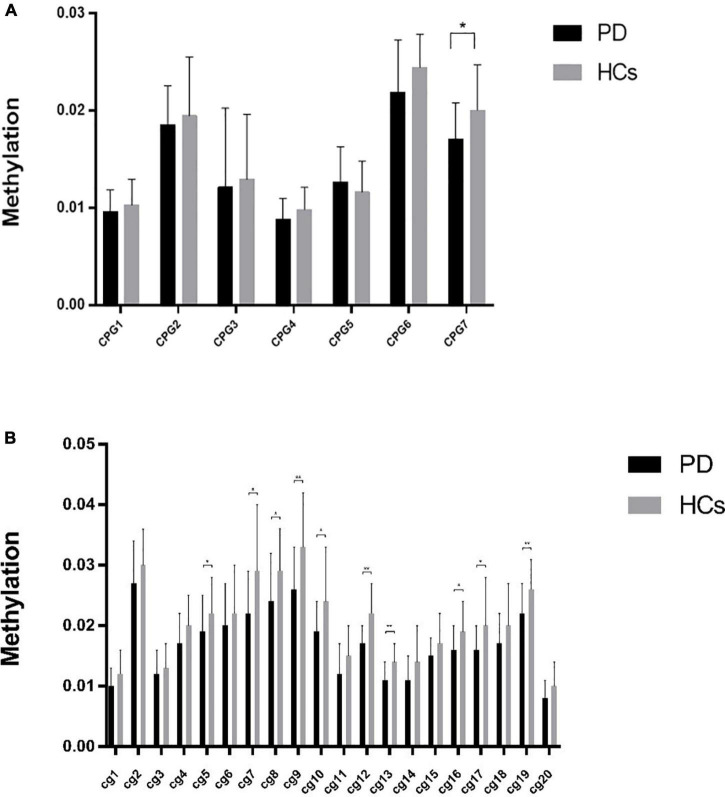
**(A)** The methylation of CPG islands in GAD1 gene in the discovery sample of patients with PD and HCs. **(B)** Lower methylation in PD compared with controls. “*” Significant at *p* < 0.05; “**” significant at *p* < 0.01.

Average methylation across CPG7 island of GAD1 gene promoter was significantly lower in patients compared to healthy controls (*p* = 0.02) (Figure 1A). There were 20 cg sites in CPG7 region and of them, the *t*-test identified 10 (5, 7–10, 12, 13, 16, 17, 19) differential cg sites that displayed significantly lower methylation (Figure 1B). These 10 cg sites were analyzed for correlation with clinical symptoms.

**FIGURE 2:**
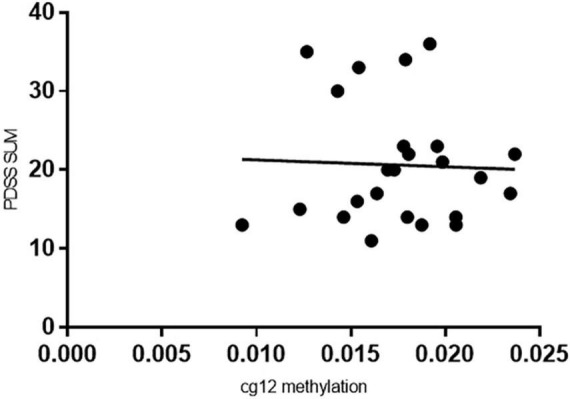
Correlation between PDSS and GAD1 cg 12 methylation. Higher PDSSSUM scores were associated with relatively decreased cg171674146 GAD1 CPG7 island methylation (*r* = –0.456, *p* = 0.025) in the total sample of 24 patients.

Pearson correlation analysis found a significant negative association between cg 12 site and clinical severity (*n* = 24, *r* = −0.456, *p* = 0.025) (Figure 2). No significant differences were found in other sites.

### Whole-Brain Differences in Gray Matter Volumes in Patients With Panic Disorder Versus Healthy Controls

First, whole-brain differences in GMV between PD patients and HCs were examined. Significant group differences (50 or more voxels, *p* < 0.05) are identified in Table 2. The largest GMV increases associated with panic disorder were in a left hemisphere cluster encompassing the superior medial frontal gyrus (SFGmed) and middle temporal gyrus (MTG). Additional differences were detected in the right inferior frontal gyrus of the orbital region (ORBinf) and the right pars opercularis. The reverse contrast (HCs > PD) identified clusters in bilateral THA, and regions of the left parahippocampal gyrus (PHG), left supramarginal gyrus (SMG), STG, superior parietal gyrus (SPG), and superior temporal gyrus of pole (TPOsup).

**TABLE 2:** Gray matter volume (GMV) abnormalities associated with panic disorder (PD).

Brain areas	Laterality	MINI coordinates			Voxels in cluster	*t*
		*x*	*y*	*z*		
SFGmed	L	0	60	34.5	106	8.339
ORBinf	R	54	45	−10.5	95	8.170
MTG	L	−70.5	−45	−12	94	7.075
IFGoperc	R	63	18	10.5	56	7.400
THA	R	18	−16.5	10.5	1040	−9.868
THA	L	−15	−24	6	942	−9.067
PHG	L	−18	−21	−19.5	163	−11.300
SMG	L	−60	−48	33	154	−8.252
STG	L	−61.5	−48	21	154	−7.306
SPG	R	16.5	−58.5	69	112	−8.556
TPOsup	R	31.5	12	−30	77	−8.851

*p < 0.05; k = 50; N = 26 (17 PD, 9 HCs); Clusters surviving whole brain correction are indicated as follows: p < 0.05; Clusters are listed in order of descending size; coordinates refer to the voxel with the peak t-value in the cluster. SFGmed, Frontal_Sup_Media; ORBinf, Frontal_Inf_Orb, MTG, Temporal_Mid; IFGoperc, Frontal_Inf_Oper; THA, Thalamus; PHG, ParaHippocampal; SMG, SupraMarginal; STG, Temporal_Sup; SPG, Parietal_Sup; TPOsup, Temporal_Pole_Sup. The largest GMV increases associated with PD were in a left hemisphere cluster encompassing the SFGmed and MTG. Additional differences were detected in right inferior frontal gyrus of orbital region, and right pars opercularis. The reverse contrast (HCs > PD) identified clusters in bilateral THA, and regions of the left PHG, left SMG, STG, SPG, and TPOsup.*

### Correlation Between Panic Disorder Clinical Severity, Gray Matter Volumes, and DNA Methylation of Glutamic Acid Decarboxylase 1

**FIGURE 3:**
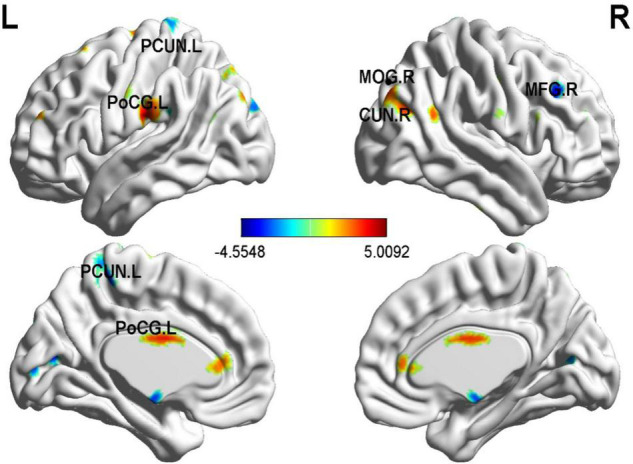
Correlation between whole-brain gray matter volume (GMV) and PDSS scores in PD patients. The left PCUN, the right middle frontal gyrus, and the right cuneus were negatively correlated with PD severity. In contrast, the right MOG, the left PoCG, the left ACG were positively correlated with PD severity. *p* < 0.01; *N* = 17.

In order to more accurately detect the effects of GMV on PD severity, we performed a multiple regression analysis and found several correlations (30 or more voxels, *p* < 0.05), as shown in Table 3 and Figure 3. The left precuneus (PCUN), the right middle frontal gyrus (MFG), and the right cuneus (CUN) were negatively correlated with PD severity. In contrast, the right middle occipital gyrus (MOG), the left PoCG, the left anterior cingulate and paracingulate gyri (ACG) were positively correlated with panic disorder severity.

**TABLE 3:** Correlation between whole-brain GMV and PDSS scores in PD patients.

Brain areas	Laterality	MINI coordinates			Voxels in cluster	*t*
		*x*	*y*	*z*		
PCUN	Left	−7.5	−37.5	60	140	−3.8653
MOG	Right	36	−82.5	42	51	3.4301
MFG	Right	33	25.5	34.5	131	−4.5548
PoCG	Left	−51	−15	27	245	3.8506
CUN	Right	12	−84	19.5	34	−3.7738
ACG	Left	0	39	9	38	3.4499

*p < 0.05; k = 30; N = 17; Clusters surviving whole brain correction are indicated as follows: p < 0.05; Clusters are listed in order of descending size; coordinates refer to the voxel with the peak t-value in the cluster. PCUN, Precuneus; MOG, Occipital_Mid; MFG, Frontal_Mid; PoCG, Postcentral; CUN, Cuneus; ACG, Cingulate_Ant. The left PCUN, the right MFG, and the right CUN were negatively correlated with PD severity. In contrast, the right MOG, the left PoCG, the left ACG were positively correlated with PD severity.*

**FIGURE 4:**
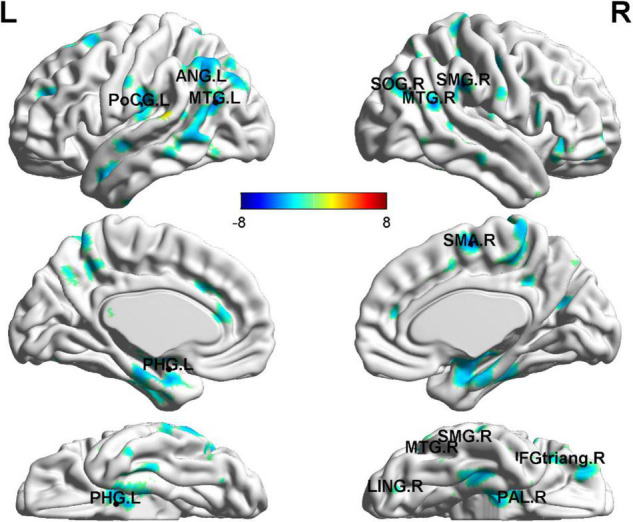
Gray matter volume abnormalities associated with cg171674146 methylation. Correlation between whole-brain GMV and cg171674146 methylation in PD patients. The multiple regression analysis showed negative correlations with the left PHG, the bilateral MTG, the left ANG, the left PoCG, and so on. *p* < 0.01; *N* = 17.

As for the correlation of cg 12 site methylation and whole-brain GMV, multiple regression analysis showed a negative correlation with the left PHG, the bilateral MTG, the left ANG, the left PoCG, and so on (Figure 4 and Table 4).

**TABLE 4:** Correlation between whole-brain GMV and cg171674146 methylation in PD patients.

Brain areas	Laterality	MINI coordinates			Voxels in cluster	*t*
		x	y	z		
PHG	Left	−12	0	−21	1178	−7.341
MTG	Left	−46.5	−66	21	391	−4.7865
SMA	Right	7.5	−6	60	169	−4.6112
LING	Right	21	−81	−3	60	−4.2328
PAL	Right	13.5	6	−4.5	160	−3.6446
MTG	Right	48	−55.5	21	355	−5.1142
ANG	Left	−42	−57	36	679	−4.2585
SMG	Right	55.5	−33	33	126	−4.2056
IFGtriang	Right	40.5	33	1.5	68	−3.7796
PoCG	Left	−57	−16.5	19.5	141	−3.3132
SOG	Right	22.5	−75	25.5	113	−4.6351

*p < 0.05; k = 50; N = 17; Clusters surviving whole brain correction are indicated as follows: p < 0.05; Clusters are listed in order of descending size; coordinates refer to the voxel with the peak t-value in the cluster. PHG: Parahippocampal; MTG: Temporal_Mid; SMA: Supp_Motor_Area; LING: Lingual; PAL: Pallidum; ANG: Angular; SMG: SupraMarginal; IFGtriang: Frontal_Inf_Tri; PoCG: Postcentral; SOG: Occipital_Sup. The multiple regression analysis showed negative correlations with the left PHG, the bilateral MTG, the left ANG, the left PoCG, and so on.*

### Gray Matter Volumes as a Mediator of the Relationship Between Glutamic Acid Decarboxylase 1 Methylation and Greater Panic Disorder Severity

Of the regions included in the analysis, GMV of 2 regions (PoCG, ANG) were found to significantly mediate the relationship between cg171674146 (cg12) methylation and PD severity.

**FIGURE 5:**
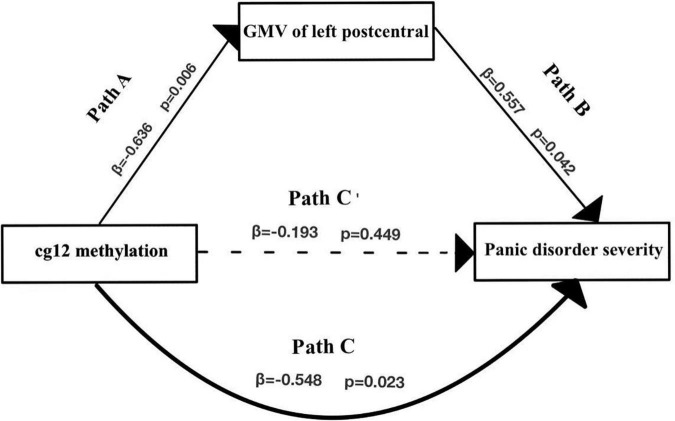
Mediation effect model with three variables in the model. Mediation analysis suggested that cg12/171674146 CpG methylation mediated the relationship between GMV in the left PoCG and PD severity. Indirect effect of cg171674146 on PDSSSUM = 95% CI: –0.873 to –0.068. Direct effect of cg171674146 on PDSSSUM (Path C′) = 95% CI: –0.619 to 0.298. Total effect of cg171674146 on PDSSSUM (Path C) = 95% CI: –0.813 to –0.157.

As shown in Figure 5 and Table 5, the cg12 methylation level had a significant predictive effect on the clinical severity of PD patients (β = −0.548, *t* = −2.537, *p* < 0.05), while the direct predictive effect of cg12 gene methylation level on the clinical severity of PD patients was not significant when mediating variables were put in (β = −0.193, *t* = −0.779, *p* > 0.05). cg12 methylation level had a significant negative predictive effect on left PoCG gray matter volumes (β = −0.636, *t* = −3.194, *p* < 0.01), and a significant positive predictive effect of PoCG GMV on clinical severity in PD patients (β = 0.557, *t* = 2.240, *p* < 0.05). In addition, the bootstrap indirect effect was−0.354 (95% CI = −0.873, −0.068), since the CI does not include zero, indicating that the cg12 methylation level was able to predict the clinical severity of PD patients through the mediating effect of left PoCG GMV with a completed mediating effect. The direct effect (−0.194) and mediating effect (−0.354) accounted for 35.34 and 64.66% of the total effect (−0.548).

**TABLE 5:** Mediation effect model of left postcentral GMV with cg171674146 methylation in PD.

Outcome variable	Predictor variable	R	R^2^	F	β	*t*
PDSS(c)	Cg12(a)	0.696	0.485	6.590	−0.193	−0.779
	Postcentral GMV(b1)				0.557	2.240*
c	a	−0.548	0.300	6.438	−0.548	−2.537*
b1	a	0.636	0.405	10.201	−0.636	−3.194**

Cg12 methylation was negatively associated with left postcentral GMV (β = −0.636, t = −3.194, p = 0.006), and left postcentral GMV was positively associated with PD severity (β = 0.557, t = 2.240, p = 0.042). The standardized indirect effect was (-0.636)*0.557 = −0.354. The standardized direct effect was −0.193 after controlling for mediation, and the standardized total effect was −0.548. *p < 0.05, **p < 0.01, ***p < 0.001.

**FIGURE 6:**
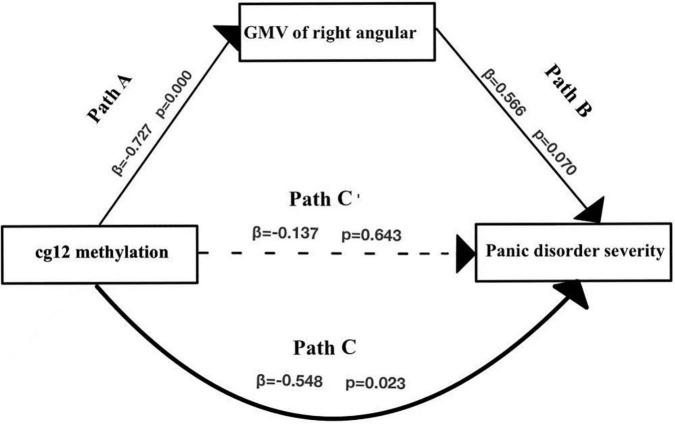
Mediation effect model with three variables. Mediation analysis suggested that cg12/171674146 CpG methylation mediated the relationship between GMV in the right ANG and PD severity. Indirect effect of cg171674146 on PDSSSUM = 95% CI: –0.983 to –0.114. Direct effect of cg171674146 on PDSSSUM (Path C′) = 95% CI: –0.652 to 0.626. Total effect of cg171674146 on PDSSSUM (Path C) = 95% CI: –0.813 to –0.157.

In the second model (Figure 6 and Table 6), when the mediating variable right ANG gray matter volumes was put into the model, the cg12 methylation level was a significant negative predictor of right ANG gray matter volumes (β = −0.727, *t* = −4.105, *p* < 0.001), and the positive predictor of right ANG GMV on the clinical severity of PD patients was not significant (β = 0.566, *t* = 1.960, *p* > 0.05). In addition, the bootstrapped indirect effect was−0.411 (95% CI = −0.983, −0.114), since the CI does not include zero, indicating that the cg12 methylation level was able to predict the clinical severity of PD patients through the mediating effect of ANG GMV. The direct effect (−0.137) and mediating effect (−0.411) accounted for 24.92% and 75.08% of the total effect (−0.548).

**TABLE 6:** Mediation effect model of right angular GMV with cg171674146 methylation in PD.

Outcome variable	Predictor variable	R	R^2^	F	β	*t*
PDSS(c)	Cg12(a)	0.672	0.451	5.750	−0.137	−0.473
	Angular GMV(b2)				0.566	1.960
c	a	0.548	0.300	6.438	−0.548	−2.537*
b2	a	0.727	0.529	16.853	−0.727	−4.105***

Right angular GMV was negatively associated with cg12 methylation (β = −0.727, t = −4.105, p = 0.000). The standardized indirect effect was (−0.727)*0.566 = −0.411. The standardized direct effect was −0.137 after controlling for mediation, and the standardized total effect was −0.548. *p < 0.05, **p < 0.01, ***p < 0.001.

## Discussion

The present study investigated whether GAD1 gene methylation impacted panic disorder severity and whether GMV played a mediating role in this relationship. Results point to a significant yet indirect relationship between GAD1 gene methylation and severity of PD. Importantly, the effects of GAD1 gene methylation on PDSS scores were mediated by gray matter volumes.

The question of whether and how GAD1 gene methylation affects PD has tremendous importance for improving our understanding of the pathogenic mechanism of panic disorder. We found that PD patients had lower gene methylation in ten GAD1 CPG sites compared to HCs. In general, DNA hypomethylation probably promotes gene expression (27). GAD1 gene hypomethylation increases GABA levels, it is possible that GAD1 hypomethylation of PD patients is not the cause of the panic behavior but a compensation for it. In view of the above results, we examined the link between methylation of ten CPG sites and PD severity. Results showed a significant negative association between methylation of cg12/171674146 site of GAD1 with clinical severity. This finding is supported by a recent study suggesting that GAD1 gene hypermethylation may play a compensatory role in panic disorder and may mediate the effect of negative life events on panic disorder (9). These results provide further evidence for the belief that GAD1 gene methylation plays an important role in the occurrence and development of PD.

The differences in GMV in the brains of PD and HCs imply that many structures are involved in the cognitive regulation of anxiety, the fear response, and the generation of threat perception, while also replicating and extending previous findings. Abnormal gray matter volumes in panic disorders has been reported in several studies (28, 29), and their results are consistent with our findings of decreased GMV in the regions of the left PHG, left SMG, left STG, right SPG, and right TPOsup, and bilateral THA in PD subjects compared to HCs. As we all know, the THA regulates emotional and cognitive functions (30). FNM suggests that changes in the temporal lobe may affect the transmission of sensory information to the THA for further filtering and subsequent “top-down” modulation of the frontal system (11). The THA interacts with temporal, parietal, subcortical limbic structures, and other FNM structures to modulate the noradrenergic system response toward fear (19, 31, 32, 33). Sensory areas of the brain, such as the occipital and temporal lobes, transmit sensory information to the FNM in order for the FNM to recognize and process facial and physical fear signals (34). Thus, the reduction of gray matter volumes in these brain regions affects the processing of fear information and the response to fear in patients with panic disorder. The results of multiple regression analysis also showed a negative correlation between cg12 site methylation with GMV of left PHG, the bilateral MTG, the left ANG, the left PoCG, and so on. Although the relationship between GAD1 gene methylation with gray matter volumes in PD remains unclear, the reduction in GABA receptor binding or reduced GABA activity occurs mainly in the frontal, limbic, temporal, and insula regions (35, 36). Consequently, anticipatory anxiety and panic attacks may be triggered by reduced GABAergic inhibition in different brain areas (37). Therefore, aberrant methylation of cg12 may be an important molecular mechanism underlying the anatomical changes in PD.

The results of the multiple regression analysis showed a significant positive correlation between right GMV of MOG, left PoCG, left anterior cingulate, and paracingulate gyri with panic disorder severity, and a negative correlation between gray matter volumes of left PCUN, the right middle frontal gyrus, and the right cuneus with PD severity. Interestingly, further mediation analysis demonstrated that GMV in the left PoCG totally mediated the association between cg12 methylation and PD severity. The PoCG has been associated with functions of receiving, integrating, and interpreting most of the sensory information in the human body (18). Abnormalities in PoCG could potentially lead to misinterpretation of somatosensory information, resulting in misleading excitatory input to the amygdala and inappropriate activation of the FNM (11). Notably, postcentral abnormalities were also seen when panic disorder was compared with MDD and generalized anxiety, suggesting that brain areas involved in somatosensory processing are more likely to be specific to patients with somatic symptoms, primarily in perceptual processing, rather than the general chronic and excessive feelings of anxiety and worry which is core features of generalized anxiety (38). Indeed, structural alterations in the PoCG may explain the specific symptoms of PD, which include abnormal perception of body signals and extreme sensation of the heartbeat. Thus, we suggest that a reduction in the volume of PoCG gray matter leads to more severe alterations in PD and this process is associated with cg12 methylation.

Path analysis showed that GMV of the ANG mediated the relationship between cg12 methylation and PD severity. The ANG plays an important role in affective regulation, empathic response, anxiety, and mood and is associated with meditation and calmness (19). In patients with panic disorder, abnormal gray matter volumes of ANG (part of parietal lobe) have been reported. For example, one study on voxel-based morphometry found reduced gray matter volumes in the parietal and temporal lobes of patients with PD (14, 15). These results provide further evidence for the idea that GMV in the right ANG potentially mediated the association between GAD1 gene methylation and PD severity.

The present study has the following limitations. First, epigenetic studies using peripheral blood do not allow for direct conclusions about the respective methylation patterns in brain tissue. Second, the study is cross-sectional and the direction of the relationship between brain structure and diseased state is uncertain. Third, the relatively small size might affect the significance of our results.

## Conclusion

In conclusion, the results of this study confirmed a significant negative relationship between cg12 methylation and PD severity. Further, we revealed that gray matter volumes of PoCG and ANG mediated this relationship, which has not been found in previous PD research. Our findings suggest that cg12 site methylation of GAD1 may affect the development of PoCG and ANG GMV and further participate in the pathophysiology of panic disorder. Future PD research should combine epigenetics with neuroimaging, which is conducive to a more comprehensive understanding of the pathogenesis of panic disorder from the epigenetic level to the neural network level.

## Data Availability Statement

The datasets presented in this study can be found in online repositories. The names of the repository/repositories and accession number(s) can be found below: NCBI, PRJNA793355.

## Ethics Statement

The studies involving human participants were reviewed and approved by the Institute of Ethics Committee of the Affiliated Brain Hospital of Nanjing Medical University. The patients/participants provided their written informed consent to participate in this study.

## Author Contributions

CW and NL designed the study and supervised the conduct of the study. GL and HD contributed to the data collection. HX, YW, SY, and YZ provided the methodological advice. HW and HX performed the data analysis and results interpretation. HW and CW drafted the manuscript, which all authors reviewed and approved for publication. All authors contributed to the article and approved the submitted version.

## Conflict of Interest

The authors declare that the research was conducted in the absence of any commercial or financial relationships that could be construed as a potential conflict of interest.

## Publisher’s Note

All claims expressed in this article are solely those of the authors and do not necessarily represent those of their affiliated organizations, or those of the publisher, the editors and the reviewers. Any product that may be evaluated in this article, or claim that may be made by its manufacturer, is not guaranteed or endorsed by the publisher.

## Footnotes

1
www.fil.ion.ucl.ac.uk/spm/
