# Supplementary material for: Glutamic Acid Decarboxylase 1 Gene Methylation and Panic Disorder Severity: Making the Connection by Brain Gray Matter Volume
Source: Front Psychiatry. 2022 May 24;13:853613. doi: 10.3389/fpsyt.2022.853613 (PMC9170964; doi:10.3389/fpsyt.2022.853613)
Supplement: Supplementary file 1 [file Data_Sheet_1.docx]

**Supplementary 1. The position of the seven CPG regions.**

| No | Target | Chr | Gene | TSS | Start | End | Length | Distance 2TSS |
| --- | --- | --- | --- | --- | --- | --- | --- | --- |
| CPG1 | GAD1_5 | 2 | GAD1 | 171673199 | 171672286 | 171672546 | 261 | -913 |
| CPG2 | GAD1_6 | 2 | GAD1 | 171673199 | 171672766 | 171672546 | 221 | -653 |
| CPG3 | GAD1_7 | 2 | GAD1 | 171673199 | 171673886 | 171673604 | 283 | 405 |
| CPG4 | GAD1_8 | 2 | GAD1 | 171673199 | 171673406 | 171673617 | 212 | 207 |
| CPG5 | GAD1_9 | 2 | GAD1 | 171673199 | 171673409 | 171673231 | 179 | 32 |
| CPG6 | GAD1_10 | 2 | GAD1 | 171673199 | 171673815 | 171674076 | 262 | 616 |
| CPG7 | GAD1_11 | 2 | GAD1 | 171673199 | 171674272 | 171674071 | 202 | 872 |

No: number, GAD1: glutamic acid decarboxylase 1, Chr: Chromosome, TSS: transcription start site, Distance 2TSS: CPG site relative distance (in bp) to TSS.

**Supplementary 2. The sites of the 20 cg in CPG7 regions.**

| No | Target | Chr | Position | Genome Position | Distance 2TSS |
| --- | --- | --- | --- | --- | --- |
| cg1 | GAD1_11 | 2 | 64 | 171674209 | 1010 |
| cg2 | GAD1_11 | 2 | 70 | 171674203 | 1004 |
| cg3 | GAD1_11 | 2 | 78 | 171674195 | 996 |
| cg4 | GAD1_11 | 2 | 86 | 171674187 | 988 |
| cg5 | GAD1_11 | 2 | 88 | 171674185 | 986 |
| cg6 | GAD1_11 | 2 | 93 | 171674180 | 981 |
| cg7 | GAD1_11 | 2 | 95 | 171674178 | 979 |
| cg8 | GAD1_11 | 2 | 104 | 171674169 | 970 |
| cg9 | GAD1_11 | 2 | 107 | 171674166 | 967 |
| cg10 | GAD1_11 | 2 | 122 | 171674151 | 952 |
| cg11 | GAD1_11 | 2 | 124 | 171674149 | 950 |
| cg12 | GAD1_11 | 2 | 127 | 171674146 | 947 |
| cg13 | GAD1_11 | 2 | 137 | 171674136 | 937 |
| cg14 | GAD1_11 | 2 | 144 | 171674129 | 930 |
| cg15 | GAD1_11 | 2 | 147 | 171674126 | 927 |
| cg16 | GAD1_11 | 2 | 153 | 171674120 | 921 |
| cg17 | GAD1_11 | 2 | 155 | 171674118 | 919 |
| cg18 | GAD1_11 | 2 | 161 | 171674112 | 913 |
| cg19 | GAD1_11 | 2 | 165 | 171674108 | 909 |
| cg20 | GAD1_11 | 2 | 174 | 171674099 | 900 |
| cg21  cg22  cg23  cg24  cg25  cg26  cg27  cg28  cg29  cg30  cg31  cg32  cg33  cg34  cg35  cg36  cg37  cg38  cg39 | GAD_10  GAD_10  GAD_10  GAD_10  GAD_10  GAD_10  GAD_10  GAD_10  GAD_10  GAD_10  GAD_10  GAD_10  GAD_10  GAD_10  GAD_10  GAD_10  GAD_10  GAD_10  GAD_10 | 2  2  2  2  2  2  2  2  2  2  2  2  2  2  2  2  2  2  2 | 26  52  56  75  93  99  111  137  143  148  155  167  173  175  177  179  197  216  223 | 171673840  171673866  171673870  171673889  171673907  171673913  171673925  171673951  171673957  171673962  171673969  171673981  171673987  171673989  171673991  171673993  171674011  171674030  171674037 | 641  667  671  690  708  714  726  752  758  763  770  782  788  790  792  794  812  831  838 |

No: number, GAD1: glutamic acid decarboxylase 1, Chr: Chromosome, Distance 2TSS: CPG site relative distance (in bp) to TSS.

**Supplementary 3. Number of subjects for each feature.**

| Feature | Patients | Controls |
| --- | --- | --- |
| Socio-demographic  PDSS  Gene Methylation  Neuroimaging  HAMA | 24  24  24  16  24 | 22  -  22  10  22 |

PDSS: Panic Disorder Severity Scale, HAMA: Hamilton Anxiety Rating Scale.
